# Ceftolozane–tazobactam- and ceftazidime–avibactam-resistant *Pseudomonas aeruginosa* mastoiditis

**DOI:** 10.1099/acmi.0.000092

**Published:** 2020-01-20

**Authors:** Jeremy Jacobs, Alexander Maris, Charles Stratton

**Affiliations:** ^1^​ Vanderbilt University Medical Center, Nashville, TN, USA

**Keywords:** antibiotic resistance, multi-drug resistance, *Pseudomonas aeruginosa*, ceftolozane–tazobactam, ceftazidime–avibactam

## Abstract

*Pseudomonas aeruginosa *is an important bacterial cause of a variety of infections and is associated with high morbidity and mortality. Infections caused by this bacterium are becoming more difficult to treat due to increasing resistance to many of the available antibiotics. Ceftolozane–tazobactam and ceftazidime–avibactam are two new cephalosporin/β-lactamase inhibitor combination antimicrobials that have demonstrated excellent *in vitro* activity against several multi-drug-resistant pathogens, including multi-drug-resistant *P. aeruginosa*. Cases of infections with isolates of multi-drug-resistant *P. aeruginosa* that are resistant to both of these antimicrobials have rarely been reported. We report a case of mastoiditis caused by *P. aeruginosa* that was resistant to both ceftolozane–tazobactam and ceftazidime–avibactam.

## Introduction

*Pseudomonas aeruginosa* is a Gram-negative bacillus that has been associated with significant morbidity and mortality, particularly in the nosocomial setting [1]. This particular bacterium has developed resistance to many of the classes of antibacterial drugs previously used to treat it [2]. As the incidence of disease caused by multi-drug-resistant isolates of this pathogen has increased, new medications have been sought out and utilized to combat these infections. Two of these new antibiotics are ceftolozane–tazobactam and ceftazidime–avibactam, second-generation β-lactam/β-lactamase inhibitor combinations that have demonstrated potent *in vitro* activity against *P. aeruginosa*, including multi-drug-resistant strains [3]. These drugs were thought to represent a backup for the treatment of multi-drug-resistant Gram-negative bacterial infections, including those caused by *P. aeruginosa*, when other medications failed or were deemed to be ineffective due to resistance. Unfortunately, the case presented here demonstrates that strains that are resistant to these newer antibiotics are already present. Additionally, it highlights the importance of preventing the spread of these infections while also continuing to develop new antibacterial compounds.

## Case report

A 59-year-old male with a past medical history significant for diabetes mellitus type II presented to our facility as a referral for chronic mastoiditis after multiple previous antibiotic regimens, as well as surgical intervention, had failed. The patient originally developed otalgia and otorrhea of his left ear 11 months prior to presenting at our facility. He was treated with multiple oral antimicrobial regimens, including oral doxycycline and oral ciprofloxacin. After several visits to an outside clinic for persistent symptoms over the course of 3 months, the patient was seen by an outside otolaryngologist (ENT), where the patient was diagnosed with a ruptured tympanic membrane. A CT scan was performed that was suggestive of chronic mastoiditis. Cultures were obtained, which grew pan-sensitive *P. aeruginosa*. The patient was prescribed CSF otic insufflation powder (combination of chloramphenicol, sulfamethoxazole and amphotericin) with improvement in the otorrhea, but continued pain and tinnitus. The patient then underwent a tympanomastoidectomy with graft placement, and intraoperative cultures grew no organisms.

A few weeks later, the patient developed upper respiratory symptoms and ruptured the graft during a coughing episode. The patient returned to the ENT complaining of otorrhea, hearing loss and tinnitus in his left ear. Cultures grew *P. aeruginosa*, this time resistant to amoxicillin–clavulanate and cefazolin. The patient was prescribed oral clindamycin and topical ofloxacin drops and instructed to follow up in 1 month. At follow-up, the patient continued to endorse otorrhea with fullness and tinnitus, and cultures grew two *P. aeruginosa* isolates, both resistant to aztreonam and ciprofloxacin, with one intermediate to gentamycin, while the other was intermediate to cefepime.

The patient was then seen by an infectious disease physician, and was treated with several weeks of meropenem, and subsequently ceftolozane–tazobactam. When the patient presented to our facility’s ENT clinic, the patient was being treated with ceftolozane–tazobactam. The patient denied any ear surgery or ear infections prior to the episode that began in July 2018.

The patient underwent a second tympanomastoidectomy with graft placement, and cultures from the surgical tissue grew *P. aeruginosa* that was resistant to all tested antimicrobials (Table 1), including ceftazidime–avibactam and ceftolozane–tazobactam, with the exception of tobramycin, which the ENT physician was hesitant to use due to the multiple ear surgeries and the risk of ototoxicity. Subsequent testing for colistin resistance demonstrated that the isolate was sensitive to colistin. Given the lack of available treatment, the patient was placed on intravenous colistin and topical tobramycin otic drops. After 3 weeks, the otorrhea persisted, as the patient was having difficulty adhering to the colistin regimen due to renal insufficiency.

**Table 1.** Antimicrobial Sensitivity Results for *P. aeruginosa* Isolate

Antimicrobial	MIC^1,2^	Interpretation^3^
Amikacin	32^1^	I^3^
Aztreonam	>16^1^	R^3^
Cefepime	>16^1^	R^3^
Ceftazidime	>16^1^	R^3^
Ceftazidime–avibactam	>16/4^2^	R^3^
Ceftolozane–tazobactam	>16/4^2^	R^3^
Ciprofloxacin	>2^1^	R^3^
Colistin	2^1^	S^3^
Gentamycin	>8^1^	R^3^
Levofloxacin	>4^1^	R^3^
Meropenem	>8^1^	R^3^
Piperacillin–tazobactam	>64/4^1^	R^3^
Tobramycin	4^1^	S^3^

^1^Minimum inhibitory concentration in micrograms per milliliter (μg ml^−1^) as determined by the BD Phoenix Automated Identification and Susceptibility Testing System manufactured by Becton, Dickinson and Company with alltesting performed according to the manufacturer’s package insert; ^2^Minimum inhibitory concentration in micrograms per milliliter (μg ml^−1^) as determined by the bioMérieux, Inc. ETEST manufactured by bioMérieux, Inc. with all testing performed according to the manufacturer’s package insert; ^3^Ranges for antimicrobial susceptibility testing and interpretations are based on [16]).

I, intermediate; R, resistant; S, sensitive.

At present, the patient continues to be treated with topical tobramycin otic drops and intravenous colistin with close monitoring of his renal and auditory function.

## Discussion

*P. aeruginosa* is a major pathogen, especially in nosocomial settings, where it is an important cause of morbidity and mortality related to urinary tract infections, pneumonia, bloodstream infections and soft tissue infections. This organism has developed resistance to many of the antibiotics typically used to treat it, including β-lactam antimicrobials such as cephalosporins and carbapenems, fluoroquinolones and aminoglycosides [4]. Studies have shown that *P. aeruginosa* is exceptionally problematic in terms of antimicrobial resistance because of its rapid ability to develop resistance and the multiple mechanisms by which it can become resistant to a variety of antimicrobials [4].

Historically, some of the most commonly used drugs to treat Gram-negative bacteria, including *P. aeruginosa*, have been β-lactam antibiotics. These antibiotics work by binding to a bacterium’s penicillin-binding protein (PBP), an enzyme that is involved in crosslinking peptidoglycan in the bacterial cell wall [5]. By inhibiting this enzyme, the bacterium is unable to complete its cell wall structure and therefore undergoes lysis, making these antibiotics bactericidal [5]. β-lactamases are enzymes produced by bacteria that hydrolyze the β-lactam ring of β-lactam antibiotics, rendering the antibiotic unable to bind to the bacterium’s PBPs, thus making them ineffective [5]. To combat these β-lactamases, antimicrobials with combination β-lactam and β-lactamase inhibitors have been developed [5]. The β-lactamase inhibitor acts to deactivate the β-lactamase enzyme, allowing the β-lactam antibiotic component to inhibit the PBPs and thereby kill the bacterium [5]. As multiple types of β-lactamase enzymes are produced by bacteria, not all β-lactamase inhibitors are capable of inhibiting all β-lactamase enzymes. In addition, as these antimicrobials have been used more frequently, resistance has become widespread.

The mechanisms by which resistance has developed include both mobile genetic elements and chromosome-based mechanisms [4]. In addition to the production of β-lactamases, including extended-spectrum β-lactamases and carbapenemases [4], other resistance mechanisms include increased expression of efflux pumps and decreased membrane permeability through the downregulation of membrane porins [6]. One particular β-lactamase is the AmpC cephalosporinase, in which increased production leads to resistance to all β-lactams except for carbapenems [4].

Two recently developed antibiotics include ceftolozane–tazobactam and ceftazidime–avibactam. Ceftolozane–tazobactam is a combination of a fifth-generation cephalosporin (ceftolozane) and a β-lactamase inhibitor (tazobactam) [7]. Ceftazidime–avibactam is a combination of a third-generation cephalosporin (ceftazidime) and a non-β-lactam β-lactamase inhibitor (avibactam) [8].

Recent studies have shown that these drugs are quite effective at treating *P. aeruginosa*, even multi-drug-resistant and meropenem-nonsusceptible strains [9, 10]. However, there are rare cases of infections with strains of *P. aeruginosa* that are resistant to at least one of these medications [11, 12]. Although a complete understanding of the resistance mechanisms to these drugs has not yet been achieved, recent evidence suggests that one mechanism for resistance to ceftolozane–tazobactam is due to mutations in the AmpC β-lactamase, leading to structural modification and overexpression of this enzyme [11]. Furthermore, a recent study has shown that ceftolozane–tazobactam is inactive against carbapenemase-producing strains of *P. aeruginosa* and a common resistance mechanism in the resistant strains was found to be a loss of the outer membrane porin D (OprD) [13].

Presumably, there are other mechanisms that could potentially lead to resistance that have not yet been characterized. As additional cases of infection with *P. aeruginosa* that is resistant to these medications occur, further investigation into elucidating these mechanisms of resistance will be beneficial for future therapeutic development.

Additional new drugs, and novel combinations of existing drugs, are currently under study [14, 15]; however, this case highlights the importance of not only continuing to develop new antimicrobials, but also to ensure the judicious and proper use of our current antimicrobial repertoire. Preventing the spread of these bacteria is paramount as multi-drug-resistant organisms are becoming ever more difficult to treat once acquired because of a limited number of effective antimicrobial agents and the systemic toxicities associated with them, as in the case presented here.
